# 淋巴浆细胞淋巴瘤/华氏巨球蛋白血症转化为弥漫大B细胞淋巴瘤5例报告并文献复习

**DOI:** 10.3760/cma.j.cn121090-20241125-00477

**Published:** 2025-09

**Authors:** 畅 周, 清洋 张, 世斌 邓, 飞跃 朱, 自勉 罗, 华 孙, 姮 李, 宏凌 彭

**Affiliations:** 1 中南大学湘雅二医院血液内科，长沙 410011 The Second Xiangya Hospital, Central South University, Changsha 410011, China; 2 永州市中心医院，永州 425000 Yongzhou Central Hospital, Yongzhou 425000, China; 3 娄底市中心医院，娄底 417000 Loudi Central Hospital, Loudi 417000, China; 4 湘潭市中心医院，湘潭 411100 Xiangtan Central Hospital, Xiangtan 411100, China; 5 岳阳市中心医院，岳阳 414000 Yueyang Central Hospital, Yueyang 414000, China

**Keywords:** Waldenstrom巨球蛋白血症, 转化, 弥漫大B细胞淋巴瘤, 临床特征, Waldenstrom macroglobulinemia, Transformation, Diffuse large B-cell lymphoma, Clinicopathology

## Abstract

**目的:**

分析淋巴浆细胞淋巴瘤/华氏巨球蛋白血症（LPL/WM）转化为弥漫大B细胞淋巴瘤（DLBCL）患者的临床特征及预后情况。

**方法:**

回顾性分析湖南省多中心自2020年12月至2023年4月期间诊治的5例LPL/WM转化为DLBCL患者的临床资料，比较转化前后的临床表现、治疗方案及疗效。

**结果:**

5例患者中男4例，女1例，中位年龄64（57～80）岁，诊断时均可见β_2_微球蛋白水平异常升高，2例合并乳酸脱氢酶升高。4例患者检测到MYD88^L265P^突变，1例携带FAT1与NOTCH1突变，5例均未检出CXCR4突变。3例患者TP53突变阴性，余2例未检测。转化前，3例患者接受布鲁顿酪氨酸激酶抑制剂治疗，1例接受BR方案治疗。所有患者均转化为非生发中心来源型DLBCL，中位转化时间为11.8（4.0～19.0）个月，多伴体重下降、淋巴结肿大、脾肿大和结外受累等表现。转化后主要采用R-CHOP方案治疗，最佳疗效为部分缓解。其中4例患者出现疾病进展，中位总生存期为16.8（10.0～26.0）个月。

**结论:**

LPL/WM向DLBCL的转化罕见，若患者出现淋巴结迅速增大和（或）新出现淋巴结受累、全身症状进行性加重、体质下降时需高度警惕转化可能。R-CHOP方案在该类患者中可取得一定缓解，但总体预后仍不理想。

## Abstract (en)

**Objective:**

To analyze the clinical features and prognosis of patients with lymphoplasmacytic lymphoma/Waldenström macroglobulinemia（LPL/WM）transformed into diffuse large B-cell lymphoma（DLBCL）.

**Methods:**

This study retrospectively analyzed the clinical data of five patients with LPL/WM transformed to DLBCL diagnosed and treated at a multicenter hospital in Hunan Province from December 2020 to April 2023. Clinical manifestations, treatment regimens, and therapeutic efficacy before and after the transformation were compared.

**Results:**

Of the five patients, four were male and one was female, with a median age of 64.0（57.0–80.0）years, all of whom had abnormally increased β_2_-microglobulin levels at diagnosis, and two were combined with increased lactate dehydrogenase levels. The MYD88^L265P^ mutation was detected in 4 patients, whereas 1 carried the FAT1 and NOTCH1 mutations, and none demonstrated CXCR4 mutations. Three patients were negative for the TP53 mutation, and two were not tested. Before transformation, three patients were treated with Bruton tyrosine kinase inhibitor therapy, and one patient was treated with the bendamustine plus rituximab regimen. All patients eventually transformed into non-growth center-derived DLBCL, with a median time to conversion of 11.8（4.0–19.0）months, and most of them presented with weight loss, lymph node enlargement, splenomegaly, and extranodal involvement. Posttransformation, the patients were mainly treated with the rituximab, cyclophosphamide, hydroxydaunorubicin, oncovin, and prednisone（R-CHOP）regimen, with an optimal outcome of partial remission. Disease progression occurred in 4 of the patients, with a median overall survival of 16.8（10.0–26.0）months.

**Conclusion:**

Transformation from LPL/WM to DLBCL is rare. Patients should remain highly vigilant for transformation if they develop rapidly enlarging lymph nodes and/or newly involved lymph nodes, worsening systemic symptoms, and declining body mass. R-CHOP regimen may induce a partial response in some cases; however, the overall prognosis remains poor.

淋巴浆细胞淋巴瘤/华氏巨球蛋白血症（LPL/WM）是一种少见的惰性成熟B细胞淋巴瘤，占非霍奇金淋巴瘤的比例不足2％[1]。其特点为由小B淋巴细胞、浆样淋巴细胞和浆细胞组成的肿瘤细胞在骨髓中浸润，并伴有分泌单克隆IgM免疫球蛋白，部分病例亦可累及淋巴结和脾。多数LPL/WM伴有MYD88^L265P^突变，需排除其他类型淋巴瘤后，方可诊断为LPL/WM[2]。尽管较为罕见，LPL/WM患者可能向弥漫大B细胞淋巴瘤（DLBCL）转化。研究显示，发生转化的患者中位总生存（OS）期显著短于未转化患者[3]。目前国内关于LPL/WM转化为DLBCL的病例报道较为有限，本文通过回顾性分析湖南省多中心5例LPL/WM向DLBCL转化患者的临床资料，旨在为临床识别转化风险并制定相应处理策略提供参考依据。

## 病例与方法

1. 病例：收集湖南省多个医疗机构2020年12月至2023年4月期间诊断的5例LPL/WM转化为DLBCL患者的临床资料（中南大学湘雅二医院、永州市中心医院、娄底市中心医院、湘潭市中心医院及岳阳市中心医院各1例），包括血常规、生化常规、心电图、骨髓形态、骨髓/淋巴结病理、免疫组化、FISH、流式细胞术、全身CT或PET-CT等资料。所有患者均符合LPL/WM的诊断标准[4]，转化后患者病理证实符合DLBCL诊断标准[5]。本研究经中南大学湘雅二医院伦理委员会批准（批件号：LYEC2024-K0264）。

2. MYD88^L265P^和CXCR4突变检测：采用Sanger测序、等位基因特异性聚合酶链反应（AS-PCR）或NGS对MYD88^L265P^及CXCR4突变进行检测。只要其中一项检测阳性，则定义为基因突变阳性。

3. 分期与评分：参照淋巴瘤分期系统Ann Arbor进行分期，转化前参照LPL/WM国际预后指数（IPSSWM）[6]进行评分及预后分层，该评分纳入5个独立预后因素：年龄>65岁、HGB≤115 g/L、PLT≤100×10^9^/L、β_2_微球蛋白（β_2_-MG）>3 mg/L、血清单克隆免疫球蛋白>70 g/L，以上各项为1分，依据这5个因素可将WM患者分为3组：低危组：0或1分且年龄≤65岁；中危组：2分或年龄>65岁；高危组：>2分。转化后参照IPI评分，该评分系统纳入5个危险因素，即年龄>60岁、美国东部肿瘤协作组（ECOG）评分≥2分、Ann Arbor分期≥Ⅲ期、结外受累部位≥2个、LDH水平异常；年龄≤60岁患者参考年龄调整的IPI（aaIPI）进行评估。

4. 疗效评估：LPL/WM疗效判定参照NCCN标准[7]，包括：完全缓解（CR）、非常好的部分缓解（VGPR）、部分缓解（PR）、微小缓解（MR）、疾病稳定（SD）、疾病进展（PD）；DLBCL按照2014年Lugano标准[8]进行疗效评估，具体分为CR、PR、SD、PD。

5. 随访：通过门诊记录、住院病历及电话进行随访。随访截止时间为2024年7月1日。随访内容包括患者距随访日期最近一次血常规、免疫球蛋白（Ig）、免疫固定电泳、疗效、有无复发、疾病转归等。无进展生存（PFS）期定义为自确诊转化至患者治疗后PD或末次随访日期的时间，OS期定义为自确诊转化至患者死亡或末次随访日期的时间。

6. 统计学处理：定量资料用*M*（范围）或*x*±*s*表示。

## 结果

1. 一般资料：5例患者中男4例，女1例，中位年龄64（57～80）岁，均无血液系统肿瘤家族史。淋巴结肿大为初诊时最常见的临床表现（4例），另有3例伴有脾肿大、1例存在髓外病灶。所有患者均未见肝肿大或冷凝集素病表现。IPSSWM评估结果显示：3例患者评分为2分，1例为3分，余1例评分不详。

5例患者中，3例在转化前出现淋巴结迅速增大和（或）新发淋巴结受累，2例出现全身症状进行性加重及体质明显下降。复查病理提示所有患者均已转化为非生发中心来源（non-GCB）型DLBCL，中位转化时间为11.8（4.0～19.0）个月。转化后，3例患者出现结外受累，分别累及骨髓、胃体及新发髂部肿块；2例伴脾肿大；2例出现体重减轻；患者均未见肝肿大。5例患者Ann Arbor分期均为Ⅲ～Ⅳ期，其中4例患者IPI评分为3分，≤60岁患者aaIPI评分为1～2分。中位随访时间为30.4（14.0～43.0）个月。

2. 实验室检查结果：转化前血常规结果显示，外周血WBC为（11.8±9.0）×10^9^/L，HGB为（91.0±7.2）g/L，PLT为（178.8±46.5）×10^9^/L，淋巴细胞绝对计数（ALC）为（8.3±8.0）×10^9^/L；白蛋白（ALB）水平偏低，为（33.6±4.2）g/L。生化检查方面，5例患者初诊时LDH为（234.2±37.3）U/L；β_2_-MG水平明显升高为（4.8±1.4）g/L；IgM为（18.3±9.3）g/L。炎症指标：C反应蛋白（CRP）为（95.3±28.7）mg/L（表1）。感染相关筛查显示：5例患者乙型病毒性肝炎表面抗原、丙型肝炎病毒抗体、梅毒螺旋体抗体及人类免疫缺陷病毒抗体均为阴性；其中3例完成EB病毒及巨细胞病毒抗体检测，结果均为阴性，另2例未检测。骨髓受累情况：转化前患者骨髓细胞学均提示淋巴细胞比例增高和（或）淋巴细胞呈多簇分布。免疫表型：5例患者的肿瘤细胞大多表达CD20、CD79、CD38和CD138；较少表达CD5及CD10。流式细胞术：4例患者可见单克隆增生的浆细胞。FISH检测结果：3例患者EB病毒编码小RNA（EBER）阴性，余2例未做；2例患者Cyclin D1阴性，余3例未检测。基因突变分析：4例患者检测到MYD88^L265P^突变，1例携带FAT1与NOTCH1突变，5例均未检出CXCR4突变。3例患者TP53突变阴性，余2例未检测。

**表1:** 5例淋巴浆细胞淋巴瘤/华氏巨球蛋白血症患者的临床特征

例号	性别	IPSSWM评分（分）	WBC（×10^9^/L）	HGB（g/L）	PLT（×10^9^/L）	ALC（×10^9^/L）	β_2_-MG（g/L）	LDH（U/L）	IgM（g/L）	MYD88^L265P^突变	CXCR4突变	是否接受治疗	疗效
1	男	2	26.4	95.0	247.0	20.7	3.6	236.4	20.1	+	–	是	PR
2	男	/	9.2	99.0	187.0	1.7	3.7	191.0	0.2	–	–	是	/
3	男	2	3.0	89.0	198.0	1.0	5.9	193.0	/	+	–	否	/
4	女	2	3.3	94.0	107.0	/	3.6	274.3	6.2	+	–	是	/
5	男	3	17.2	78.0	155.0	9.7	6.9	276.3	28.7	+	–	是	PR

**注** IPSSWM：华氏巨球蛋白血症国际预后评分系统；ALC：淋巴细胞绝对计数；β_2_-MG：β_2_微球蛋白；LDH：乳酸脱氢酶；IgM：免疫球蛋白M；/：未检测；+：突变阳性；–：突变阴性；PR：部分缓解

转化后血常规结果显示，外周血WBC为（4.6±1.7）×10^9^/L，HGB为（93.2±15.1）g/L，PLT为（113.2±61.7）×10^9^/L，ALC为（1.0±0.5）×10^9^/L；ALB水平偏低，值为（35.4±2.7）g/L。5例患者LDH为（654.3±618.4）U/L；β_2_-MG水平升高，值为（3.7±0.7）g/L；IgM水平异常增高，值为（13.7±20.4）g/L。炎症指标：CRP为（50.4±28.8）mg/L（表2）。组织病理检查结果：5例患者病理组织切片均提示转化为non-GCB型DLBCL，生发中心标志物CD10表达阴性。免疫组化提示肿瘤细胞大多表达CD20、Pax-5、Mum-1。

**表2:** 5例淋巴浆细胞淋巴瘤/华氏巨球蛋白血症患者转化为弥漫大B细胞淋巴瘤后的临床特征

例号	Ann Arbor分期	IPI评分（分）	aaIPI评分（分）	WBC（×10^9^/L）	HGB（g/L）	PLT（×10^9^/L）	ALC（×10^9^/L）	β_2_-MG（g/L）	LDH（U/L）	IgM（g/L）	ALB（g/L）	CRP（mg/L）
1	Ⅳ期	3	2	2.8	68.0	59.0	1.0	4.0	992.6	3.4	35.6	82.4
2	Ⅲ期	3	/	5.7	104.0	98.0	1.7	4.6	169.0	/	36.1	56.0
3	Ⅲ期	/	1	7.0	108.0	225.0	/	/	125.0	49.0	30.9	59.5
4	Ⅳ期	3	2	4.8	102.0	57.0	/	3.3	1 718.1	1.8	39.3	/
5	Ⅳ期	3	/	2.6	84.0	127.0	0.3	2.8	266.7	0.8	35.2	3.7

**注** IPI：国际预后指数；aaIPI：年龄调整的国际预后指数；ALC：淋巴细胞绝对计数；β_2_-MG：β_2_微球蛋白；LDH：乳酸脱氢酶；IgM：免疫球蛋白M；ALB：白蛋白；CRP：C反应蛋白；/：未检测

3. 治疗与转归：转化前，5例LPL/WM患者中有4例接受治疗。其中3例接受以布鲁顿酪氨酸激酶抑制剂（BTKi）为主的持续治疗，中位治疗时间为14.0（4.0～21.0）个月；1例患者接受5个周期的BR（苯达莫司汀+利妥昔单抗）方案及1个周期的ZR（泽布替尼+利妥昔单抗）方案治疗。2例患者获得PR，余2例患者疗效不详。

5例患者转化后均接受了系统性治疗，初始均优先选用淋巴瘤一线方案，其中部分因不耐受或治疗相关血液学毒性逐步调整为二线或以上治疗方案，具体治疗方案详见表3。5例患者的最佳治疗反应均为PR，对R-CHOP方案的应答率较好；在接受二线及以上方案的患者中，治疗应答率达100％。随访期间，4例患者出现PD，其中2例因PD死亡。5例患者的中位PFS期为11.5（5.0～23.0）个月，中位OS期为16.8（10.0～26.0）个月。转化前接受治疗的4例患者，中位OS期为13.0（9.0～19.0）个月；而1例在未接受任何治疗前即确诊转化的患者，OS期为23.0个月，且至随访结束时尚未出现PD。

**表3:** 5例淋巴浆细胞淋巴瘤/华氏巨球蛋白血症患者转化为弥漫大B细胞淋巴瘤后的治疗及预后

例号	治疗方案	疗效	疾病进展	结局
1	6个周期ZR-CHOP方案	PR	是	10个月后死亡
2	9个周期R-CHOP方案	PR	是	19个月后死亡
3	1个周期R-CHOP方案；5个周期奥布替尼+R-CHOP方案	PR	否	存活
4	2个周期伊布替尼+R-CHOP方案；1个周期ZBR方案；3个周期R+低剂量Hyper-CVAD/MA方案	PR	是	存活
5	2个周期Z-CHOP方案；9个周期Z+ mini CHOP方案；1个周期VEN+GemOx方案，VEN+低剂量GDP方案	PR	是	存活

**注** ZR-CHOP：泽布替尼、利妥昔单抗、环磷酰胺、阿霉素、长春新碱及泼尼松；ZBR：泽布替尼、苯达莫司汀及利妥昔单抗；R+低剂量Hyper-CVAD/MA：利妥昔单抗+环磷酰胺、长春新碱、阿霉素、地塞米松与高剂量甲氨蝶呤及阿糖胞苷交替；VEN + GemOx：维奈克拉+吉西他滨及奥沙利铂；VEN +低剂量GDP：维奈克拉+吉西他滨、地塞米松、顺铂或卡铂；PR：部分缓解

## 讨论

LPL/WM是一种罕见的惰性B细胞淋巴瘤，特点为骨髓及其他器官中蓄积分泌IgM的恶性淋巴细胞[9]。随着研究深入，LPL/WM患者发生继发性恶性肿瘤（SM）的现象逐渐明确，其风险较一般人群高49％，多见于急性髓系白血病（AML）和侵袭性淋巴瘤[10]。Wang等[11]研究发现，中国发生SM的LPL/WM患者OS期明显缩短，其中AML的发生多与治疗相关，而侵袭性淋巴瘤则可能源于转化。自1967年首次报道LPL/WM向更具侵袭性的淋巴瘤转化以来，类似病例不断增多[12]。转化类型以DLBCL最为常见，也可转化为免疫母细胞淋巴瘤、外周T细胞淋巴瘤及霍奇金淋巴瘤等[13]。

尽管各研究中转化发生率不一，但数据显示[14]，随着患者的疾病存活期延长，转化风险亦增加。两项来自美国大型中心的研究显示5年、10年和15年累积转化发生率分别为1％、2％、4％及2％、5％、6％[14]。治疗因素可能与转化风险相关。传统治疗多以烷化剂为主，后逐渐采用核苷类似物，如氟达拉滨。研究提示，核苷类似物可能增加转化风险[15]，使用烷化剂和核苷类似物者在6年内转化发生率分别为11％与8％[16]。近年来，随着治疗手段的进步，如BR和DRC（地塞米松+利妥昔单抗+环磷酰胺）方案，患者预后显著改善[4]。但BR方案在中位随访30个月时转化或骨髓异常发生率为8％，DRC方案在随访7个月时为5％[17]。随着对LPL/WM本质认识加深，其B细胞起源特性明确，抗浆细胞治疗逐步被替代。以伊布替尼为代表的BTKi成为重要靶向治疗药物，但其对转化风险的影响尚不明确[18]。基因组学为个体化治疗提供方向。MYD88基因型在转化风险评估中具有价值：MYD88^WT^型患者转化风险为15％，而MYD88^L265P^突变者仅为1％，其10年累积发生率分别为20％和1％[2]。

LPL/WM向DLBCL的转化相对罕见，中位转化时间为5.36年[19]。本研究中5例转化患者中位转化时间为11.8（4.0～19.0）个月，转化时间较短可能为患者症状轻微、就诊延迟所致。临床上，若患者出现淋巴结迅速增大、新发淋巴结受累或全身症状加重，应高度警惕转化。组织活检为诊断金标准，可由临床表现或^18F^-氟代脱氧葡萄糖（FDG）PET-CT引导活检部位[20]。转化后DLBCL多为non-GCB型，缺乏CD10表达，预后较差[21]。Durot等[22]提出的转化Waldenström国际预后指数（tWIPI）评分系统基于LDH升高、PLT<100×10^9^/L及既往治疗史，定义三类风险组（低危、中危、高危），对应2年生存率分别为81％、47％与21％。本研究中，例1、4、5为高危，例2为中危，例3为低危，与实际病程基本一致，提示该评分对中高危患者可能需要更多预测指标以优化判断。转化后最常用治疗方案为R-CHOP方案，亦可采用R-（mini）CHOP、R-ACVBP或R-CVP等方案。一线治疗总体缓解率达61％，R-CHOP方案应答良好[23]。转化后患者中位OS期为16.0个月，死亡原因主要为疾病进展或感染。对诱导化疗有反应的患者，可考虑auto-HSCT作为巩固治疗[24]。初步研究表明CD19 CAR-T细胞治疗对转化DLBCL有效，且无意外不良反应，但仍需更大规模验证[25]。

本研究存在局限：首先，样本量较小，限制了统计效能。其次，仅2例患者完成免疫球蛋白重链可变区（IGHV）重排检测以确认转化前后淋巴瘤克隆同源性。尽管其余3例未进行该项检测，但结合以下证据可推测其为同源克隆转化：明确的LPL/WM病史，且转化后DLBCL预后差，与文献报道特征一致[21]；转化后病灶与原LPL/WM受累部位重叠；DLBCL均为non-GCB亚型，免疫表型与LPL/WM一致，部分保留原有突变并伴新突变，提示克隆演进。尽管如此，未进行分子水平验证仍可能存在偏倚，未来需扩大样本并系统开展基因追踪研究。

综上，LPL/WM是一种惰性淋巴瘤，转化虽罕见，但预后显著恶化。转化后虽可对治疗应答良好，但生存期受限。因此，早期识别与干预转化至关重要。目前有关转化的研究有限，亟需更多研究以明确机制、开发新疗法，提高缓解率与延长生存期。
